# DO FRIENDS GET UNDER THE SKIN? DAILY INTERACTIONS AND CARDIOVASCULAR FUNCTIONING AMONG BLACK AND WHITE AMERICANS

**DOI:** 10.1093/geroni/igac059.1028

**Published:** 2022-12-20

**Authors:** Yee To Ng, Sae Hwang Han, Karen Fingerman, Kira Birditt

**Affiliations:** UT Austin, Ann Arbor, Michigan, United States; University of Texas at Austin, Austin, Texas, United States; The University of Texas at Austin, Austin, Texas, United States; University of Michigan, Ann Arbor, Michigan, United States

## Abstract

Scarce research has examined racial differences in cardiovascular health in the context of social interactions. This study investigated whether (a) friend interactions were associated with better cardiovascular functioning, and (b) such associations vary among Black and White adults. This study employed dual assessment techniques—ecological momentary assessments (EMA) and ambulatory physiological assessments—to examine the co-occurrence of social interactions and cardiovascular functioning. Multilevel models revealed no racial differences in ambulatory HRV and frequency of friend interactions throughout the day. Findings revealed a between-person link of friend interactions and better HRV in the overall sample. Race-stratified models found a within-person link between friendship interaction and worse HRV and a between-person link between friendship interaction and better HRV for Blacks but not for Whites. Findings suggest friend interactions are more salient for Blacks’ cardiovascular health and may serve as a modifiable factor for preventing cardiovascular diseases.

